# On the epidemiology of influenza: reply to Radonovich et al

**DOI:** 10.1186/1743-422X-6-121

**Published:** 2009-08-11

**Authors:** John J Cannell, Michael Zasloff, Cedric F Garland, Robert Scragg, Edward Giovannucci

**Affiliations:** 1 Department of Psychiatry, Atascadero State Hospital, 10333 El Camino Real, Atascadero, CA 93423, USA; 2 Departments of Surgery and Pediatrics, Georgetown University, Washington, DC, USA; 3 Department of Family and Preventive Medicine, University of California San Diego, La Jolla, CA, USA; 4 Department of Epidemiology and Biostatistics, University of Auckland, Auckland, New Zealand; 5 Departments of Nutrition and Epidemiology, Harvard School of Public Health, Boston, MA, USA

## Abstract

On the epidemiology of influenza: reply to Radonovich LJ, Martinello RA, Hodgson M, Milton DK, Nardell EA. Influenza and ultraviolet germicidal irradiation. Virol J. 2008, 5:149

## Commentary

To the Editor:

We thank Radonovich *et al *[1] for commenting on our paper [2], in which we attempted to use the epidemiology of vitamin D to clarify the manifold mysteries surrounding the epidemiology of influenza. Since our publication, Ginde *et al *[3] have produced additional evidence in support of our theories. They studied the relationship between 25-hydroxy-vitamin D [25(OH)D] levels and recent upper respiratory tract infections (URI) in 18,883 participants in the Third National Health and Nutrition Examination. Compared to individuals with serum 25-hydroxy-vitamin D levels of > 30 ng/ml, those with levels < 10 ng/ml had 55% higher odds of a recent URI. Furthermore, very recent evidence indicates 25(OH)D levels of even 30 ng/ml often signify chronic substrate starvation [4], thus the full antimicrobial properties of vitamin D may be understated.

Radonovich *et al *did not supply any evidence against the main hypotheses proposed in our paper, only to a speculation we made about ultraviolet germicidal air irradiation. They take factual issue with our theory that the ultraviolet C radiation (UVC) lamps, used in the past to sterilize the upper air in some VA hospitals, may have exerted some, or most, of their effects – not by sanitizing air – but by increasing 25(OH)D levels.

Radonovich *et al *assert there was no patient exposure from UVC germicidal lamps, as they were installed to irradiate only the upper air and never shone directly on patients, thus "minimizing UV exposure in the occupied space below." Careful inspection of such an arrangement, in a 1957 Baltimore VA hospital, is illuminating [5]. Photographs show lights that seemed to shine indirectly on patients, apparently 24 hours per day, seven days a week. Depending on the characteristics of the reflective surfaces, it seems possible – even likely from the photographs – that a small amount of UVC was reflected downward toward the patients.

Radonovich *et al *then assert that even if some UVC reflected downward, it could not produce Vitamin D, as UVC radiation does not do so, citing MacLaughlin et al [6]. The belief that UVC radiation cannot produce vitamin D may be a widespread misconception. Fortunately for humans, as UVC is highly carcinogenic, UVC does not penetrate the atmosphere and certainly does not trigger cutaneous Vitamin D during the course of normal human affairs. However, closer reading of their MacLaughlin *et al *reference [6] would have revealed that significant photosynthesis of vitamin D from 7-dehydrohydrocholesterol (7-DHC) occurs at UVC wavelengths in the epidermal lipids the authors extracted [as illustrated in MacLaughlin *et al's *figure 1(B)]. Indeed, per photon, UVC photosynthesis is greater than UVB [as illustrated in MacLaughlin *et al's *figure 1(C)].

Furthermore, several animal studies indicate that UVC, which should never be used in man, is highly effective in both producing vitamin D and in treating rachitic rats [7]. Knudson and Benford studied numerous UV wavelengths in rats, finding UVC as effective as any of the UVB wavelengths studied in curing rickets [8]. If all human Vitamin D production is intra-epidermal, the academic question appears to be, how deep does UVC penetrate human epidermis? Campbell *et al *found evidence that significant amounts of UVC penetrate through the stratum corneum, stratum lucidum, stratum granulosum, and small amounts of UVC even reach the upper layers of the 7-DHC-rich stratum spinosum [9]. Thus, UVC penetrates far enough into the epidermis to trigger some intra-epidermal vitamin D production. Again, because UVC is so mutagenic, we are heartened to find no studies that directly test this theory in living humans.

However, even if no UVC penetrated the stratum corneum, Helmer and Jensen published a remarkable human/animal study in 1937, showing that significant amounts of Vitamin D are made on the surface of human skin [10]. They collected surface oils from young men, irradiated the oils, and showed those oils rapidly cured rachitic rats. Then, they tested a very practical and important question, can those oils be removed by washing. Indeed they found a simple water wash removed much of the Vitamin D from the surface of human skin. Holick *et al's *landmark study showing most human Vitamin D production occurs in the deep epidermis was based on surgically obtained (and assumedly surgically prepped) skin samples that then had surface oils removed again by washing in hot water [11]. Indeed, to accurately address the question, one would need to obtain unwashed human skin, difficult to do even from cadavers.

It appears to us that the percentage of Vitamin D made on the surface of the human epidermis, compared to that made inta-epidermally, is unknown at this time and in need of additional and careful research. What percentage of the Vitamin D made in human skin after sun exposure is removed by simply washing? Furthermore, as the percentage made of the surface is significant, studies of cutaneous Vitamin D production in modern humans, unless unwashed, will not give accurate estimates of Vitamin D production in early man and thus an estimate of the "natural" 25(OH)D levels present when the human genome evolved.

We repeat our aside hypothesis that the patients in UVC irradiated hospitals may have been the beneficiaries of more than just cleaner air. Much more importantly, influenza is just one of many seasonal infections sensitive to the broad spectrum anti-microbial peptides (AMP) that vitamin D up-regulates [2]. Invasive pneumococcal disease, meningococcal disease, and group A streptococcal disease are all highly seasonal [12-14] and all are sensitive to AMP [15-17]. Would vitamin D be an effective adjuvant in these devastating diseases?

As research into vitamin D's remarkable effects on innate immunity quickens [18], we hope to see the day when infectious disease experts use all available antimicrobial strategies, including testing the serum 25(OH)D level in all patients with severe infections. In our opinion, physicians treating such patients should vigorously replete them, quickly achieving 25(OH)D levels in the high range of normal.
